# Supplementary material for: Dogs fail to reciprocate the receipt of food from a human in a food-giving task
Source: PLoS One. 2021 Jul 14;16(7):e0253277. doi: 10.1371/journal.pone.0253277 (PMC8279367; doi:10.1371/journal.pone.0253277)
Supplement: S1 Appendix — (DOC) [file pone.0253277.s011.doc]

# **Dogs fail to reciprocate the receipt of food from a human in a food-giving task**

## **Jim McGetrick^1,2^*, Lisa Poncet^1,3,4^, Marietta Amann^1^, Johannes Schullern-Schrattenhofen^1^, Leona Fux^1^, Mayte Martínez^1^, Friederike Range^1^**

^1^Domestication Lab, Konrad Lorenz Institute of Ethology, Department of Interdisciplinary Life Sciences, University of Veterinary Medicine, Vienna, Ernstbrunn, Austria

^2^Institute of Animal Welfare Science, Department for Farm Animals and Veterinary Public Health, University of Veterinary Medicine, Vienna, Austria

^3^Normandie Université, Unicaen, CNRS, EthoS (Éthologie animale et humaine) – UMR 6552, Caen, France

^4^Université de Rennes, CNRS, EthoS (Éthologie animale et humaine) – UMR 6552, Rennes, France

*Corresponding author: Jim.McGetrick@vetmeduni.ac.at

**Study 1**

**Full model for each analysis**

**Proportion of times the subjects pressed the button**

full = glmer(cbind(presses,no_press)~condition*human_type + z.day_order + z.condition_order + human_experience_order + (1 + condition.SFC + condition.T + human_type.U + z.day_order + z.condition_order || subject_ID) +

(1 + condition.SFC*human_type.U + condition.T*human_type.U + z.condition_order + human_experience_order.U || human_ID) +

(1+condition.SFC + condition.T + z.condition_order||dyad) +

(1|observation), family = binomial, data = xdata, control=contr)

**Latency to approach the human**

full = coxph(Surv(start_time, lat_prox, event) ~ human_type + z.day_order + human_experience_order + frailty(human_ID), data = xdata)

**Duration of proximity to the human**

full = glmmTMB(tr.prop_dur~human_type + z.day_order + human_experience_order + (1|subject_ID) + (1|human_ID), family = beta_family(link="logit"), data = test.data)

**Study 2**

**Full model for each analysis**

**Proportion of times the subjects pressed the button**

full = glmer(cbind(presses,no_press)~condition*human_type + z.day_order + z.condition_order + (1 + condition.SFC + condition.T + human_type.U + z.condition_order + z.day_order || subject_ID) +

(1 + condition.SFC*human_type.U + condition.T*human_type.U + z.condition_order || human_ID) + (1 + z.condition_order ||dyad) + (1|observation), family = binomial, data = xdata, control = contr)

**Latency to approach the human**

full = coxph(Surv(start_time, lat_prox, event) ~ human_type + z.day_order + frailty(human_ID), data = xdata)

**Duration of proximity to the human**

full = glmmTMB(tr.prop_dur~human_type + z.day_order + (1|subject_ID) + (1|human_ID), family = beta_family(link="logit"), data = test.data)

**Gazing at the helpful human in the experience phase**

**Methods**

**Coding**

The duration of gaze at the helpful human in experience phase sessions in study 1 was coded. Instances of looking at or seeing any part of the human’s body (e.g. seeing the human’s hand on the button) were coded. Coding for gaze duration was divided among five experimenters and for interobserver reliability, all five experimenters coded the same 20% of the videos. Six trials from one session with one subject were missing due to the absence of a video recording.

**Statistical analysis**

For each trial we determined the proportion of time spent gazing at the helpful human in the 3 second period immediately before button pressing and in the 3 second period immediately after button pressing. For one single trial with one subject, there were less than 3 seconds on video prior to button pressing; therefore, the total duration used to determine the proportion was adjusted.

To determine whether trial number had an influence on the proportion of time subjects spent looking at the helpful human before button pressing, we fitted a Generalized Linear Mixed Model (GLMM) with a beta error distribution and a logit link function [1–3]. As such beta models cannot handle zeroes and ones, and our dataset comprised both, we transformed the response using the following formula (where “x” is the variable to be transformed; see Smithson and Verkuilen [3]):

x’ = x.(length(x)-1) + 0.5

length(x)

We included the fixed effects of trial and trial squared. We included random intercept effects of subject and human, and random slopes of trial and trial squared were included within both.

We fitted the model using the function “glmmTMB” from the package “glmmTMB” (version 1.0.2.1 [4]). Prior to fitting the model, and prior to squaring trial number, we z-transformed trial number to a mean of zero and a standard deviation of one to allow for an easier interpretation of results and to ease model convergence. All correlations between random slopes and random intercepts were removed due to convergence issues. The model was not overdispersed (dispersion parameter: 0.6989). A full-null model comparison was carried out, as an overall test of the effect of trial number and trial number squared on the proportion of time spent looking at the helpful human before button pressing.

Confidence intervals of model coefficients were derived using 1,000 parametric bootstraps using the function “simulate” of the package “glmmTMB” (version 1.0.2.1 [4]) and a wrapper kindly provided by Roger Mundry. Tests of the individual fixed effects were derived with likelihood ratio tests [5] by using the R function “drop1” and setting the argument “test” to “Chisq”.

The effect of trial on the duration of gaze at the human after button pressing was analysed using an identical model structure; however, no random slopes were included due to convergence issues. This model was not overdispersed (dispersion parameter: 0.8290). The sample for both models included a total of 1,044 observations across 21 subjects and 12 humans. Interobserver reliability for duration of gaze at the helpful human before and after button pressing was moderate (before: ICC = 0.725, n_observations_ = 193, n_raters_ = 5, *P* < 0.001; after: ICC = 0.61, n_observations_ = 193, n_raters_ = 5, *P* < 0.001). Models were plotted using R (version 4.0.2 [6]).

**Results**

**Proportion of time spent gazing at the human before button pressing**

Overall, there was a significant effect of the fixed effects on the proportion of time spent gazing at the helpful human before button pressing (full-null model comparison: χ^2^ = 11.437, *df* = 2, *P* = 0.003). More specifically, with a greater number of trials, the proportion of time spent gazing at the helpful human increased (see **S1 Table** and **S1 Fig**).

**S1 Table** Results of the model analysing the effects of trial and trial squared on the proportion of time subjects spent gazing at the helpful human before button pressing in the experience phase. Estimates are presented along with standard errors, confidence intervals, and likelihood ratio test results.

| Term | Estimate | SE | Lower CI | Upper CI | χ^2^ | *df* | *P* |
| --- | --- | --- | --- | --- | --- | --- | --- |
| Intercept | -0.794 | 0.214 | -1.209 | -0.391 | - | - | - |
| Trial^a^ | 0.290 | 0.084 | 0.117 | 0.460 | 8.792 | 1 | 0.003 |
| Trial squared^a^ | -0.084 | 0.046 | -0.174 | 0.010 | 2.668 | 1 | 0.102 |

^a^Trial was z-transformed to a mean of 0 and a standard deviation of 1 prior to inclusion in the model and prior to squaring.

**Proportion of time spent gazing at the human after button pressing**

Overall, a trend was revealed for the influence of the fixed effects on the proportion of time spent gazing at the helpful human after button pressing (full-null model comparison: χ^2^ = 5.569, *df* = 2, *P* = 0.062). There was a weak effect whereby the proportion of time spent gazing at the human increased with more trials (see **S2 Table** and **S2 Fig**).

**S2 Table** Results of the model analysing the effects of trial and trial squared on the proportion of time subjects spent gazing at the helpful human before button pressing in the experience phase. Estimates are presented along with standard errors, confidence intervals, and likelihood ratio test results.

| Term | Estimate | SE | Lower CI | Upper CI | χ^2^ | *df* | *P* |
| --- | --- | --- | --- | --- | --- | --- | --- |
| Intercept | -2.310 | 0.126 | -2.572 | -2.083 | - | - | - |
| Trial^a^ | 0.065 | 0.032 | 0.002 | 0.130 | 4.019 | 1 | 0.045 |
| Trial squared^a^ | 0.043 | 0.036 | -0.026 | 0.111 | 1.408 | 1 | 0.235 |

^a^Trial was z-transformed to a mean of 0 and a standard deviation of 1 prior to inclusion in the model and prior to squaring.

**References**

1. Cribari-Neto F, Zeileis A. Beta regression in R. J Stat Softw. 2010;34(2):1–24.

2. Grün B, Kosmidis I, Zeileis A. Extended beta regression in R: Shaken, Stirred, Mixed, and partitioned. J Stat Softw. 2012;48(1):1–25.

3. Smithson M, Verkuilen J. A better lemon squeezer? Maximum-likelihood regression with beta-distributed dependent variables. Psychol Methods. 2006;11(1):54–71.

4. Brooks ME, Kristensen K, van Benthem KJ, Magnusson A, Berg CW, Nielsen A, et al. glmmTMB balances speed and flexibility among packages for zero-inflated generalized linear mixed modeling. R J. 2017;9(2):378–400.

5. Barr DJ, Levy R, Scheepers C, Tily HJ. Random effects structure for confirmatory hypothesis testing: Keep it maximal. J Mem Lang. 2013;68(3):255–78.

6. R Core Team. R: A language and environment for statistical computing. Vienna, Austria: R Foundation for Statistical Computing; 2020.
